# Have there been efforts to integrate malaria and schistosomiasis prevention and control programs? A scoping review of the literature

**DOI:** 10.1371/journal.pntd.0011886

**Published:** 2024-01-24

**Authors:** Claudia Duguay, Sydney Raduy, Engluy Khov, Natacha Protopopoff, Cindy Feng, Alison Krentel, Manisha A. Kulkarni

**Affiliations:** 1 School of Epidemiology and Public Health, University of Ottawa, Ottawa, Canada; 2 London School of Hygiene & Tropical Medicine, London, United Kingdom; 3 Department of Community Health & Epidemiology, Dalhousie University, Halifax, Canada; 4 Bruyère Research Institute, Ottawa, Ontario, Canada; RTI International, UNITED STATES

## Abstract

Malaria and schistosomiasis are two important parasitic diseases that are a particular threat to young children and pregnant women in sub-Saharan Africa. Malaria and schistosomiasis prevention and control strategies primarily focus on the distribution of long-lasting insecticidal nets and the delivery of praziquantel tablets to at-risk populations in high burden settings through mass drug administration, respectively. The objective of this scoping review was to identify previous efforts to integrate malaria and schistosomiasis prevention and control programs in the literature and to summarize the strategies and approaches used in these programs following the PRISMA-ScR guidelines. We reviewed published and grey literature using a combination of keywords and search terms following themes surrounding “malaria”, “*Plasmodium falciparum”*, “*Anopheles*”, “schistosomiasis”, “*Schistosoma haematobium”*, “*Schistosoma mansoni”*, *and* “snails”. Neither a date limit nor relevant terms for prevention and control were used. Out of 6374, eight articles were included in the scoping review—three articles investigated the integration of mass drug administration for schistosomiasis with the administration of antimalarials, four articles investigated the effect of administering antimalarials on malaria, schistosomiasis, and their co-infection, and one article assessed the impact of an educational intervention on malaria and schistosomiasis knowledge and preventative behaviors. Our findings suggest that there is an opportunity to link disease control programs to increase access and coverage of interventions to improve outcomes for malaria, schistosomiasis, and their co-infection. Further research is needed on the potential benefits, feasibility, and cost-effectiveness of integrating malaria and schistosomiasis prevention and control programs.

## Author summary

Malaria and schistosomiasis are two diseases that are preventable, treatable, and affect the most vulnerable populations in the world—in particular, young children in rural areas of sub-Saharan Africa. These two diseases overlap geographically, co-infect vulnerable populations, share common risk factors, and have the potential for integrated control strategies. The objective of this scoping review was to identify previous efforts to integrate malaria and schistosomiasis prevention and control programs in the literature and to summarize the strategies and approaches used in these programs. Eight articles were included in the scoping review–three articles investigated the integration of mass drug administration for schistosomiasis with the administration of antimalarials, four articles investigated the effect of administering antimalarials on malaria, schistosomiasis, and their co-infection, and one article assessed the impact of an educational intervention on malaria and schistosomiasis knowledge and preventative behaviors. Our findings suggest that there is an opportunity to link disease control programs to increase access and coverage of interventions to improve outcomes for malaria, schistosomiasis, and their co-infection.

## Introduction

Malaria and schistosomiasis are two preventable and treatable parasitic diseases that are a particular threat to young children under 14 years old and pregnant women in sub-Saharan Africa [1–5]. Malaria is transmitted by *Anopheles* mosquitoes and is caused by five different *Plasmodium* parasite species, with *P*. *falciparum* being the predominant species in sub-Saharan Africa [1]. There are two forms of schistosomiasis in sub-Saharan Africa caused by two different *Schistosoma* species–*S*. *mansoni* causes intestinal schistosomiasis and *S*. *haematobium* causes urogenital schistosomiasis.

There are three important tools to control and prevent malaria which are responsible for most of the decline in the number of malaria cases and deaths between 2000 and 2015—including long lasting insecticidal nets (LLIN), indoor residual spraying, and treatment with artemisinin-based combination therapies [1,6]. Globally, there were an estimated 247 million malaria cases in 2021 across 84 countries, which was a 7% increase since the establishment of the World Health Organizations (WHO’s) Global Technical Strategy for malaria in 2015 [1,7]. The Global Technical Strategy was developed to set targets to reduce malaria incidence and mortality rates by at least 90% compared to the 2015 baseline, yet there have been challenges in reducing the number of malaria cases and deaths since its establishment. This is likely due to biological threats (pyrethroid resistance in LLINs, antimalarial drug resistance), a reduced coverage of LLINs, and further challenges as a result of the COVID-19 pandemic (disruptions in malaria testing and LLIN distributions)–all of which points to the need for sustained efforts and new control strategies [1,6–8].

Other malaria measures such as preventative chemotherapy have been recommended by WHO since 2022 to reach vulnerable populations of various age groups [9]. In moderate-to high transmission settings with perennial or seasonal transmission, the WHO recommends the use of intermittent preventative treatment in pregnancy and in school-aged children (IPTp and IPTsc), irrespective of infection status, to treat existing infections and prevent new infections of malaria [9]. For children belonging to age groups at high risk of severe malaria in areas of perennial and seasonal transmission of malaria, the WHO recommends perennial or seasonal malaria chemoprevention, respectively (PMC and SMC) [9]. PMC, formerly intermittent preventative treatment in infants, has extended its target age group to all children at risk for severe malaria [9]. SMC differs from PMC in that it is administered in periods of greatest risk of malaria in areas with seasonal transmission [9].

The WHO recommends the delivery of preventative chemotherapy to at-risk populations, at a frequency that depends on the endemicity of the community, as a prevention and control tool for five neglected tropical diseases, including schistosomiasis [2,10,11]. For example, the delivery of praziquantel tablets is targeted to all age groups, including children over the ages of two, pregnant women after the first trimester, and lactating women, once a year in areas with schistosomiasis prevalence of ≥10%. In many countries, mass drug administration (MDA) for schistosomiasis relies on the donation of praziquantel tablets to be distributed in endemic communities through school and community health structures. Between 2011 and 2020, Merck KGaA donated 1.26 billion praziquantel tablets for the prevention and control of schistosomiasis [12]. Despite these efforts, 236 million people across 51 countries still require preventive chemotherapy for schistosomiasis in 2021 [10].

While praziquantel is a valuable tool for treating and preventing schistosomiasis, it does not address the underlying social and ecological determinants. Although more resource intensive and logistically complex, snail control with molluscicides and providing safe water and sanitation are critical components to break the schistosomiasis transmission cycle and reduce the need for treatment in at-risk populations [13–16].

Where malaria and schistosomiasis are co-endemic, co-infection is likely to occur. Afolabil et al. (2022), published a systematic review of 16 articles investigating malaria and schistosomiasis co-infections in children living in endemic countries, and a meta-analysis of raw extracted data from the included articles [17]. This systematic review found that malaria and schistosomiasis co-infection ranged from 0.2% in one study in Tanzania to 62.9% in Mali, with a pooled co-infection prevalence of 19.2% [17–20]. Although the co-infection in the Pwani region of Tanzania was low (n = 2/992, 0.2%), there were only two children in the study who were infected with schistosomiasis, both of whom were also co-infected with malaria [19]. Given the potential for co-infection in endemic areas, there is an opportunity for local integrated control strategies for malaria and schistosomiasis, such as combining activities (i.e., IPTsc for malaria and MDA for schistosomiasis) to optimize the use of existing resources as well as increase intervention access and/or coverage for an improved outcome. There is currently a proof-of-principle modeling tool published by Standley et al. 2018, designed for local public health officials or policy makers to provide guidance on how and when to integrate malaria and schistosomiasis control methods–however, only for LLIN distribution, indoor residual spraying, and schistosomiasis MDA [21]. There is also evidence of other types of integration. For instance, MDAs for soil transmitted helminth (STH) and schistosomiasis are frequently combined since children are often co-infected and the simultaneous administration of praziquantel and albendazole is safe [22]. There is also evidence of successful integration of a prevention strategy, which included LLIN distribution with an MDA for lymphatic filariasis, another neglected tropical disease, for the control of malaria and lymphatic filariasis [23]. The objective of this scoping review is to identify previous efforts to integrate malaria and schistosomiasis prevention and control programs and to summarize the strategies and approaches used in these programs. To our knowledge, a review identifying the available evidence for integrating malaria and schistosomiasis prevention and control programs has not yet been conducted.

## Methods

To conduct and report this scoping review, we followed the PRISMA-ScR (Preferred Reporting Items for Systematic Reviews and Meta-Analysis extension for Scoping Reviews) (S1 File: PRISMA-ScR Checklist) [24,25]. The detailed published protocol is available on Protocol.io [26].

A systematic search of three academic databases (Medline (Ovid), EMBASE, and Web of Science) was conducted on August 17, 2022. A detailed search strategy for each database was designed and piloted in consultation with a librarian at the University of Ottawa to identify the optimal combination of keywords used. We examined the available electronic databases using combination searches of the following terms: “malaria” OR “*Plasmodium falciparum”* OR “*Anopheles*” AND “schistosomiasis” OR “*Schistosoma haematobium”* OR “*Schistosoma mansoni”* OR “snails”. Detailed search strategies and terms used for each database are reported elsewhere [26]. To ensure a comprehensive search of all relevant articles, key terms for prevention and control were excluded from the electronic database searches, and two stages of article screening were conducted with incremental inclusion criterions with each stage. All identified articles were imported into COVIDENCE, a systematic review management software, to screen and manage the results of the search [27].

The first stage of the review involved two of the three reviewers (CD, SR, ELK) independently identifying potentially relevant articles based on information provided in the title and abstract. Articles were included if 1) they addressed malaria and schistosomiasis, and 2) evaluated or described an intervention or control method for malaria and schistosomiasis (Table 1). Articles were also included if the information provided in the title and abstract was not sufficient to determine if it met the inclusion criteria.

**Table 1:** Inclusion and exclusion criteria.

Key Theme	Screening Phase 1 (Title and abstract review)	Screening Phase 2 (Full text review)
**Malaria and schistosomiasis**	1. Address malaria and schistosomiasis	1. Address malaria, schistosomiasis, and their co-infection
**Integrated prevention and control**	1. Evaluate or describe an intervention or control method for malaria and schistosomiasis	Evaluate or describe joint delivery of malaria and schistosomiasis prevention or control activity (i.e., educational program) Evaluate or describe a common platform to deliver integrated malaria and schistosomiasis intervention (i.e., mass drug administration for schistosomiasis and LLIN distribution for malaria)
**Study Type**		Primary research or protocol for primary research Not a laboratory-based study
**Language**		1. Published in English or in French

The second stage of the review involved at least two of the three reviewers independently identifying relevant publications based on information provided in the full article. Studies were included if they 1) addressed malaria, schistosomiasis, and their co-infection, 2) evaluated or described a joint delivery of malaria and schistosomiasis prevention or control activity (i.e., educational program) or evaluated or described a common platform to deliver an integrated malaria and schistosomiasis intervention (i.e., mass drug administration for schistosomiasis and IPTsc in schools), 3) reported on primary research or protocol for primary research, and 4) were published in French or in English. Articles were excluded if they were a laboratory-based study. Any discordance in the process was discussed among all three reviewers.

The grey literature search was conducted after the completion of the peer-review literature search. A systematic search of four grey literature databases (Google search engine, WHO Institutional Repository for Information Sharing search engine, clinical trial registry (https://clinicaltrials.gov), and targeted website search) and forward and backward citation search of the included articles were conducted between December 16, 2022, and January 4, 2023. Detailed search strategies and terms used for each database can be found as a supplementary file (S2 File: Search Strategy). The first 25 hits for each search in Google and WHO search engine (as sorted by relevance) were screened. Where abstracts were not available, tables of contents were reviewed, followed by full-text screening. Microsoft Excel was used to manage the grey literature search.

From the included articles (academic and grey literature search), one reviewer (CD) extracted data from the articles following a pre-specified extraction sheet (S3 File: Extraction Sheet). Extracted data included descriptive elements of the study (first author, year of publication, study period, study type, year of program implementation, country of program implementation), and program/intervention characteristics (target population, program/intervention objectives, program/intervention type, key findings, and items from the Template for Intervention Description and Replication (TIDieR) checklist). TIDieR is a 12-item checklist that includes the brief name, why, what (materials), what (procedure), who provided, how, where, when and how much, tailoring, modifications, how well (planned), how well (actual) of a program [28].

## Results

**Fig 1:**
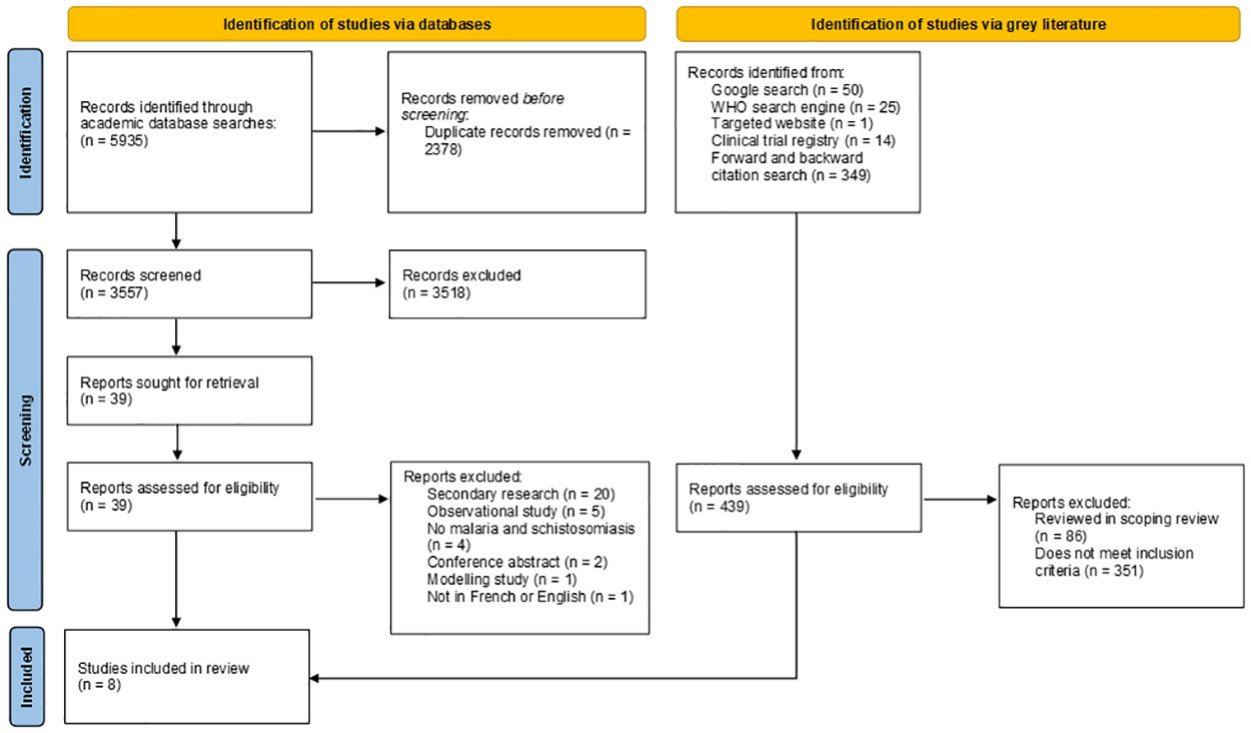
Preferred reporting items for systematic reviews and meta-analyses extension for scoping reviews (PRISMA-ScR) checklist adapted from the PRISMA 2020 statement [25].

6374 citations were generated from the electronic database searches on Medline (Ovid) (n = 1864), EMBASE (n = 2874), Web of Sciences (n = 1197), Google Search (n = 50), WHO Search engine (n = 25), clinical trial registry (n = 14), forward and backward citation search (n = 349), and targeted website (n = 1) for which only eight articles were included in the scoping review (Fig 1). A complete list of all screened studies can be found as a supplementary file (S4 File: Complete Data File). Characteristics of the included articles are summarized in Table 2. The eight articles were published in English from 1988–2022. The target populations were predominantly pre-school-aged children and school-aged children, with two studies also targeting adults. The interventions outlined in the articles were carried out in sub-Saharan Africa and based in schools (n = 3), health centres (n = 2), schools and health centres (n = 1), or were not specified (n = 2). The eight interventions fit into one of the following categories: 1) integration of MDA and the administration of antimalarials, 2) antimalarials to treat malaria and schistosomiasis co-infection, or 3) an educational program.

**Table 2:** Summary of included studies.

Authors	Country	Age of Study population	Type of intervention	Disease targeted	Citation
Midzi et al. (2011)	Zimbabwe	5–17 years old	MDA (praziquantel, albendazole) and antimalarials (chloroquine, sulphadoxine and pyrimethamine) Educational program	Malaria, schistosomiasis, and STH	[29]
Cohee et al. (2018)	Malawi	5–15 years old	MDA (praziquantel, albendazole) and antimalarials (artemether-lumefantrine) Educational program	Malaria, schistosomiasis, and STH	[30]
Afolabi et al. (2022)	Senegal	1–14 years old	MDA (praziquantel) and antimalarials (amodiaquine and sulphadoxine-pyrimethamine)	Malaria and schistosomiasis	[31]
Ekeh & Adeniyi (1988)	Nigeria	Second year of high school	Educational program	Malaria. Schistosomiasis, dracunculiasis, and onchocerciasis	[32]
Boulanger et al. (2007)	Senegal	15–74 months	Antimalarials (artesunate-pyrimethamine) to treat co-infection	Malaria and schistosomiasis	[33]
Abay et al. (2012)	Ethiopia	5 years and older	Antimalarials (artemether-lumefantrine) to treat co-infection	Malaria and schistosomiasis	[34]
Zoleko-Manego et al. (2022)	Gabon	All ages	Antimalarials (artesunate-pyronaridine) to treat co-infection	Malaria and schistosomiasis	[35]
Adedoja et al. (2015)	Nigeria	1–15 years old	Antimalarials (artemether-lumefantrine) to treat co-infection	Malaria and schistosomiasis	[36]

**Abbreviations**: MDA: mass-drug administration; STH: soil-transmitted helminthiasis

### Integration of MDA and the administration of antimalarials

Three articles examined the integration of school-based MDAs and the administration of antimalarials [29–31]. Midzi et al. (2011), aimed to reduce malaria and schistosomiasis co-infection by combining the schistosomiasis and STH MDA with an educational campaign for malaria, schistosomiasis, and STH–specifically to recognize the signs of malaria for prompt health seeking behavior [29]. A cohort of school-aged children were followed for nearly three years and were tested for urinary schistosomiasis using urine filtration methods, intestinal schistosomiasis using the Kato Katz method, and malaria using thick smears of slides from venous blood [29]. Schistosomiasis was only tested during the follow-up visit, but children were encouraged to seek prompt malaria treatment between visits if they experienced any malarial-like symptoms [29]. A child was only treated with praziquantel (schistosomiasis) or chloroquine, sulphadoxine and pyrimethamine (malaria) when they tested positive for either respective disease [29]. This study found that, after 33-months, administering praziquantel at school and sustained prompt malaria treatment significantly reduced the prevalence of malaria, schistosomiasis, and their co-infection by 50.6% (8.1% to 4.0%), 12.9% (31.7% to 27.6%), and 80.2% (13.1% to 2.6%), respectively [29].

Cohee et al. (2018), assessed the participation, safety, and tolerability of adding antimalarial treatment and an educational program to an existing schistosomiasis and STH combined MDA [30]. The education campaign was student-led and was used to increase knowledge about the symptoms, modes of transmission, and prevention for malaria, schistosomiasis, and STH; and to promote participation in the MDA [30]. Unlike Midzi et al. (2011), they distributed artemether-lumefantrine, praziquantel, and albendazole irrespective of infection status to all students in the school [30]. Overall, the enhanced MDA with antimalarials and the educational program was well accepted and did not result in more adverse events compared to the scheduled MDA program [30].

Unlike the two other studies, the article by Afolabi et al. 2022, is a protocol for a randomized control trial evaluating the safety and effectiveness of delivering schistosomiasis MDA through the SMC platform for children in Senegal [31]. Children will be allocated to either the SMC-only group (amodiaquine and sulphadoxine-pyrimethamine) or SMC and MDA group (amodiaquine, sulphadoxine-pyrimethamine, and praziquantel), and the authors will assess adverse reaction for each intervention arm as well as the prevalence and intensity of co-infection [31]. There will also be a qualitative component which includes structured interviews to assess the acceptability, feasibility, enablers, and barriers of combining these two interventions [31].

### Antimalarials to treat malaria and schistosomiasis co-infection

Boulanger et al. (2007), Abay et al. (2012), Zoleko-Manego et al. (2022), and Adedoja et al. (2015), investigated the effect of administering antimalarials on malaria, schistosomiasis, and their co-infection [33–36]. These studies investigated the efficacy of two different antimalarial combinations (artesunate-pyrimethamine and artemether-lumefantrine) and found them both to be effective for treating malaria and schistosomiasis [33–36]. In the studies by Boulanger et al. (2007) and Zoleko-Manego et al. (2022), only confirmed cases of malaria were tested for urinary schistosomiasis and treated with artesunate-pyrimethamine [33,35]. This combination of antimalarial was found to be effective with cure rates of 56% [35] and 92.6% [33] after four weeks. In the studies by Abay et al. (2012), and Adedoja et al. (2015), only confirmed cases of malaria were tested for intestinal schistosomiasis and urogenital schistosomiasis and treated with artemether-lumefantrine [34,36]. This combination of antimalarial was also found to be effective with cure rates of 100% for intestinal and urogenital schistosomiasis after four weeks [34,36].

### Educational program

Ekeh & Adeniyi (1988), investigated the impact of an educational intervention on the prevention of malaria and schistosomiasis, and other neglected tropical diseases (onchocerciasis and dracunculiasis) [32]. This four-day education campaign focused on educating school-aged children on the causes, prevention, and treatment of these diseases while emphasizing changes in behavior to mitigate disease exposure [32]. In this study, the authors found that when knowledge was supported by enabling (i.e., environmental) and reinforcing factors (i.e., someone’s ability to sustain or maintain a change in behavior), changes in behaviors can occur [32].

## Discussion

This scoping review identified different types of integrated prevention and control programs for malaria and schistosomiasis that were effective in reducing co-infection prevalence and yielded additional benefits. The integrated strategies included: 1) school-based MDA for schistosomiasis along with the administration of antimalarials for malaria; 2) the use of antimalarials to treat malaria and schistosomiasis; and 3) an educational campaign. The studies included in this scoping review emphasize the importance of multi-sectoral approaches for each disease—including an educational campaign to mitigate disease exposure along with a pharmaceutical intervention to prevent and control infection in target populations [29,30,32]. This review highlights that the integration of prevention and control programs goes beyond these two diseases, and can include other diseases that are co-endemic with similar intervention strategies (i.e., STH, onchocerciasis, dracunculiasis) [29,30,32].

Progress towards malaria and schistosomiasis elimination will have a positive effect on multiple Sustainable Development Goals (SDGs), including but not limited to SDG 3.3 that states “by 2030, [we should] end the epidemics of AIDS, tuberculosis, malaria and neglected tropical diseases and combat hepatitis, water-borne diseases and other communicable diseases” [37,38]. The SDGs recognize the importance and need for integrated approaches and solutions such as working together across sectors, government, organizations, and disciplines (among others) to achieve a common goal and to build a more sustainable and resilient future [39,40]. Researchers and agencies have also highlighted the opportunity of integrating disease control activities for neglected tropical diseases, including schistosomiasis, with one of the more funded “big three” (malaria, HIV/AIDS, and tuberculosis) programs, however little research has been done to provide evidence for such integration [41–43]. For instance, a WHO publication reviewed in the grey literature search (but excluded from the scoping review) noted the possibility to integrate malaria and schistosomiasis control programs by integrating snail control with malaria vector control, but did not provide concrete recommendations or supporting evidence [2].

Where malaria and schistosomiasis are co-endemic, co-infection is likely to occur. Given the shared population at risk, there is an opportunity to leverage and capitalize on existing infrastructures and resources to optimize current prevention and control programs for malaria and schistosomiasis. For example, three studies investigated the integration of MDA and the administration of antimalarials in schools, recognizing the potential to coordinate efforts in a single venue (schools) to an at-risk population (school-aged children) [29–31]. The other five studies investigated the administration of one intervention to address both diseases (pharmaceutical and educational) [32–36]. An important feature to note is that 88% (7/8) of the interventions outlined in this scoping review include a pharmaceutical intervention. Although artemisinin-based combination treatments and praziquantel tablets are still efficacious in treating malaria and schistosomiasis, respectively, the heavy reliance on these treatments raises concerns for the potential emergence of drug resistance [1,2].

There is evidently a missed opportunity for other types of integrated efforts, including non-pharmaceutical interventions such as the provision of safe water, sanitation, and hygiene (WASH) that could result in a significant reduction in exposures to malaria and schistosomiasis. There are twelve major diseases associated with inadequate WASH, including malaria and schistosomiasis, that account for 3.3% of the total deaths (approximately 2 million people) globally [44]. Access to safe water and sanitation facilities plays a pivotal role in both the transmission and prevention of malaria and schistosomiasis. For malaria, better management of water sources eliminate the accumulation of standing and stagnant water which can serve as mosquito larval breeding sites [44]. For schistosomiasis, the transmission cycle requires direct contact with water contaminated with cercaria. These water sources, typically unimproved (i.e., unprotected springs, surface water [45]), are contaminated when an infected individual either practices open defecation/urination near/in a water source or an infected individual uses an unimproved sanitation facility (i.e., pit latrines without slab [45]) that does not properly keep the sewage out of the environment. Therefore, interventions that address safe WASH could have a positive effect on both malaria and schistosomiasis–yet we did not find any studies that investigated this type of intervention.

The majority of the studies in this scoping review (n = 5) also state that the integration of schistosomiasis and malaria prevention and control programs have the potential to be cost-effective, but fail to provide any definition or breakdown of such costs [29–31,33,34]. The tablets for school-based schistosomiasis MDAs are largely donated by pharmaceutical companies, and the cost for delivering such programs is approximately $0.50 USD per child (reflecting the cost of delivering the tablets rather than the tablets themselves) [2]. As for malaria, the total funding for its prevention and control fell short of the 2021 targets to remain on track for the Global Technical Strategy, for the third consecutive year [1]. With more than 247 million malaria cases and 236 million people requiring preventative chemotherapy for schistosomiasis, now, more than ever, there is a need and opportunity to link control programs to increase access and coverage for improved outcome, while reducing intervention costs and combat donor fatigue [1,10].

This review has several potential limitations. First, some relevant documents may have been excluded unintentionally from this scoping review, but this risk was mitigated by reviewing both academic and grey literature databases. Although the grey literature search did not contribute many sources in this scoping review (n = 2), it did highlight a knowledge gap and ongoing work in the field (i.e., an active randomized controlled trial investigating the safety and effectiveness of delivering MDA and IPTsc as well as a non-randomized study published three months after the initial electronic database search investigating the effectiveness of artemisinin-based combination therapy on malaria, schistosomiasis, and their co-infection [31,35]). The majority (88%, n = 44) of the custom Google searches were also research articles that either did not meet the inclusion criteria (n = 11) or were already included in the scoping review search results (n = 33), which highlights the ongoing research and focus on advancing the evidence for integrating malaria and schistosomiasis prevention and control strategies. These ongoing efforts could lead to concrete guidelines and recommendations. A second limitation is that scoping reviews are exploratory in nature and are meant to address broad questions such as identifying the type of efforts made to integrate malaria and schistosomiasis prevention and control programs [46,47]. Although the scope was wide, it is evident and clear from this scoping review that while there is a need to move away from siloed approaches for disease prevention, more evidence is needed to inform such policies.

## Conclusion

It is imperative to build resilient, and sustainable programs to maintain efforts for the control and elimination of malaria and schistosomiasis. To date, this is the first scoping review that identifies previous efforts to integrate malaria and schistosomiasis programs. Future research priorities should include additional randomized control trials that combine malaria and schistosomiasis activities, such as the one being conducted by Afolabil et al. (2022), to inform the integration of programs targeting these two diseases [31]. Non-pharmaceutical interventions (i.e., access to safe WASH and the impact of nutritional supplementation) should also be investigated further to assess their effectiveness on reducing malaria and schistosomiasis infection. Despite associations between safe WASH and nutritional supplementation and mono-infections with malaria and schistosomiasis, no studies were identified in this scoping review likely because these outcomes were not investigated in the same article. The different types of interventions outlined in this review highlight the need to identify key stakeholders for each disease prevention and control program, and to understand local contexts to establish the feasibility of integrating malaria and schistosomiasis prevention and control programs. Such research can support malaria and schistosomiasis program stakeholders to work together in the critical window of opportunity to reach the 2030 targets outlined by the SDGs, Global Technical Strategy, and WHO 2030 neglected tropical disease roadmap.

## Supporting information

S1 File PRISMA-ScR checklist. (DOCX)

S2 File Search strategy. (DOCX)

S3 File Extraction sheet. (DOCX)

S4 File Complete data file. (XLSX)
