# Supplementary material for: Have there been efforts to integrate malaria and schistosomiasis prevention and control programs? A scoping review of the literature
Source: PLoS Negl Trop Dis. 2024 Jan 24;18(1):e0011886. doi: 10.1371/journal.pntd.0011886 (PMC10807771; doi:10.1371/journal.pntd.0011886)
Supplement: S2 File — (DOCX) [file pntd.0011886.s002.docx]

**Grey Literature Search Strategy**

**Table 1.** Search results – Custom Google search engines

| **#** | **Search** | **# Results** | **# Results screened** | **# Results retained** |
| --- | --- | --- | --- | --- |
| 1 | malaria AND schistosomiasis | ~ 1,830,000 | 25 | 0 |
| 2 | falciparum AND schistosoma | ~ 309,000 | 25 | 0 |

**Table 2.** Search results – Custom advanced World Health Organization Institutional Repository for Information Sharing search engine

| **#** | **Search** | **# Results** | **# Results screened** | **# Results retained** |
| --- | --- | --- | --- | --- |
| 1 | malaria schistosomiasis | 417 | 25 | 0 |
| Sorted by relevance | | | | |

**Table 3.** Websites identified through targeted web searches

| **#** | **Search** | **# Results** | **# Results screened** | **# Results retained** |
| --- | --- | --- | --- | --- |
| 1 | malaria schistosomiasis | 6 | 6 | 0 |
| 2 | falciparum schistosoma | 8 | 8 | 0 |

**Table 4.** Websites identified through targeted web searches

| **#** | **Website name/organization** | **Link** |
| --- | --- | --- |
| 1 | TDR | https://tdr.who.int/ |
